# Bis(*N*-methyl-*N*-phenyl­carbamo­yl)disulfane

**DOI:** 10.1107/S1600536812016030

**Published:** 2012-04-28

**Authors:** Alayne L. Schroll, Maren Pink, George Barany

**Affiliations:** a Department of Chemistry, Saint Michael’s College, Colchester, Vermont 05439, USA; b Department of Chemistry, Indiana University, Bloomington, Indiana 47408, USA; c Department of Chemistry, University of Minnesota, Minneapolis, Minnesota 55455, USA

## Abstract

The title compound, C_16_H_16_N_2_O_2_S_2_, has been synthesized by several different high-yield routes, and has been encountered as a co-product in a number of reaction pathways, ever since it became of inter­est to our research program over 30 years ago. We now confirm the proposed mol­ecular structure in which the mol­ecule exhibits a twofold axis of symmetry through the mid-point of the S—S bond and the two planes defined by the (carbamo­yl)sulfenyl moieties are essentially perpendicular to each other [dihedral angle = 81.55 (14)°].

## Related literature

For the preparation of the title compound, and of very closely related chemical structures, see: Kobayashi *et al.* (1973 ▶); Barany *et al.* (1983 ▶); Schroll & Barany (1986 ▶); Schrader *et al.* (2011 ▶). For related structures, see: CSD refcodes BOWGAV (Bereman *et al.*, 1983 ▶), DBZOSS01&03 (Rout *et al.*, 1983 ▶; Paul & Srikrishnan, 2004 ▶), METHUS03 (Wang & Liao, 1989 ▶), NELTUT (Fun *et al.*, 2001 ▶), JAXPOO (Raya *et al.*, 2005 ▶), UDALER (Li *et al.*, 2006 ▶) and EMASIV (Singh *et al.*, 2011 ▶). For the theoretical optimum torsion angle about the disulfane, see: Pauling (1949 ▶); Torrico-Vallejos *et al.* (2010 ▶) and references cited therein. 
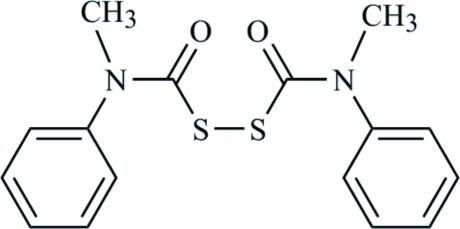


## Experimental

### 

#### Crystal data

C_16_H_16_N_2_O_2_S_2_
*M* *_r_* = 332.43
Monoclinic, 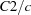

*a* = 15.286 (3) Å
*b* = 9.7849 (18) Å
*c* = 11.597 (2) Å
β = 107.433 (3)°
*V* = 1654.9 (5) Å^3^
*Z* = 4
Mo *K*α radiation
μ = 0.33 mm^−1^
*T* = 296 K
0.40 × 0.16 × 0.13 mm

#### Data collection

Bruker SMART CCD diffractometer
Absorption correction: multi-scan (*SADABS*; Bruker, 2010 ▶) *T*_min_ = 0.880, *T*_max_ = 0.958
5726 measured reflections
1468 independent reflections
1140 reflections with *I* > 2σ(*I*)
*R*_int_ = 0.031

#### Refinement

*R*[*F* ^2^ > 2σ(*F* ^2^)] = 0.037
*wR*(*F* ^2^) = 0.110
*S* = 1.05
1468 reflections
101 parameters
H-atom parameters constrained
Δρ_max_ = 0.21 e Å^−3^
Δρ_min_ = −0.18 e Å^−3^

### 

Data collection: *SMART* (Bruker, 2001 ▶); cell refinement: *SAINT* (Bruker, 2001 ▶); data reduction: *SAINT*; program(s) used to solve structure: *SHELXS97* (Sheldrick, 2008 ▶); program(s) used to refine structure: *SHELXL97* (Sheldrick, 2008 ▶); molecular graphics: *SHELXTL* (Sheldrick, 2008 ▶); software used to prepare material for publication: *SHELXTL*.

## Supplementary Material

Crystal structure: contains datablock(s) I, global. DOI: 10.1107/S1600536812016030/qm2060sup1.cif

Structure factors: contains datablock(s) I. DOI: 10.1107/S1600536812016030/qm2060Isup2.hkl

Supplementary material file. DOI: 10.1107/S1600536812016030/qm2060Isup3.cml

Additional supplementary materials:  crystallographic information; 3D view; checkCIF report

## Figures and Tables

**Fig. 1.**
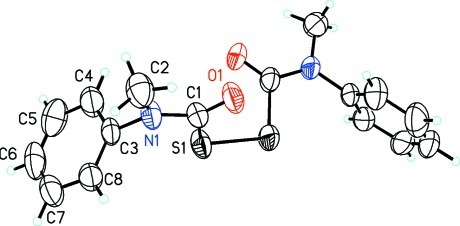
Crystallographic structure of the title compound showing 50% probability displacement ellipsoids and with all non-hydrogen atoms labelled and numbered.

## supplementary crystallographic information

### Comment

Bis(*N*-methyl-*N*-phenylcarbamoyl)disulfane (C_16_H_16_N_2_O_2_S_2_) was first reported by Kobayashi *et al.* (1973). The compound became of interest to our research program over thirty years ago (Barany *et al.*, 1983; Schroll and Barany, 1986) and has been synthesized by several different high-yield routes, as well as encountered as a co-product in a number of reaction pathways (Barany *et al.*, 1983; Schroll and Barany, 1986; Schrader *et al.*, 2011). We now confirm the molecular structure of the title compound by single-crystal X-ray analysis. The disulfane reported herein is the flagship of the homologous series of bis(*N*-methyl-*N*-phenylcarbamoyl)polysulfanes, C_16_H_16_N_2_O_2_S*_n_* , which have been prepared and structurally characterized for *n* = 1–6.

The title compound exhibits a twofold axis of symmetry through the center of the S–S bond, and all bond distances and angles are within expected ranges. The N–C bond distance is 1.35 Å, consistent with ~60% double bond character, with the consequence that the (carbamoyl)sulfenyl atoms (S1,C1,O1,N1,C2,C3) are in a plane. The aromatic ring is nearly perpendicular to the (carbamoyl)sulfenyl plane, with a torsion angle of 92.5° (C2–N1–C3–C4). The S–S bond length of 2.03 Å is slightly shorter than the 2.07 Å reported for the S–S bond length in elemental sulfur (S_8_), suggesting that some partial double bond character extends through the S–S bond due to its adjacency to carbonyl groups on both sides. Several other reference compounds also have an S–S bond length of 2.01–2.03 Å (Bereman *et al.*, 1983; Rout *et al.*, 1983; Paul and Srikrishnan, 2004; Fun *et al.*, 2001; Raya *et al.*, 2005; Li *et al.*, 2006; Singh *et al.*, 2011). The most noteworthy feature of the title compound is the torsion angle about the disulfane, which is 81.6° and as such is somewhat smaller than the theoretical optimum of 90.0° (Pauling, 1949; Torrico-Vallejos *et al.*, 2010) that has been explained as allowing for minimal mutual repulsion of pπ orbital electron lone pairs in sulfur. A comparable deviation from theory was reported for dibenzoyl disulfide (Rout *et al.*, 1983; Paul & Srikrishnan, 2004), where the torsion angle is 80.8°. Bis(*N*-methyl-*N*-phenylthiocarbamoyl)disulfane, which only differs from the title compound by two thiocarbonyls in place of two carbonyls, has a torsion angle about the disulfane of 89.8° and shows a conformation that is not completely superimposable on the title compound (Fun *et al.*, 2001).

*Note regarding nomenclature:* The title compound is named in a manner that is consistent with our prior publications. The closely related C_16_H_16_N_2_S_4_ was named bis(*N*-methyl-*N*-phenylthiocarbamoyl) disulfide by Fun *et al.* (2001), but we have chosen the "disulfane" revised name for consistency.

Table 1 Selected geometric parameters (Å, °)

N1–C1 1.345 (3)

N1–C2 1.461 (3)

N1–C3 1.442 (2)

C1–O1 1.209 (2)

C1–S1 1.825 (2)

S1–S1 2.0262 (11)

C2–N1–C3–C4 92.5 (3)

C1–S1–S1–C1 81.55 (14)

Symmetry operator (*a*): -*x* + 1, *y*, -*z* + 1/2

### Experimental

The title compound was prepared in high yield from the reaction of *N*-methylaniline with bis(chlorocarbonyl)disulfane, and recrystallized from hot carbon tetrachloride/chloroform (3:2) in 60–85% recovery or from hot acetone in 75% recovery (Barany *et al.*, 1983).

### Refinement

H atoms were positioned geometrically and refined using a riding model with C—H = 0.93 Å (aromatic) or 0.96 Å (methyl), and *U*_iso_(H) = 1.2*U*_eq_(C) for aromatic and 1.5*U*_eq_(C) for methyl H atoms.

### Crystal data

**Table:**

C_16_H_16_N_2_O_2_S_2_	*F*(000) = 696
*M**_r_* = 332.43	*D*_x_ = 1.334 Mg m^−^^3^
Monoclinic, *C*2/*c*	Mo *K*α radiation, λ = 0.71073 Å
*a* = 15.286 (3) Å	Cell parameters from 1966 reflections
*b* = 9.7849 (18) Å	θ = 2.5–24.4°
*c* = 11.597 (2) Å	µ = 0.33 mm^−^^1^
β = 107.433 (3)°	*T* = 296 K
*V* = 1654.9 (5) Å^3^	Needle, colorless
*Z* = 4	0.40 × 0.16 × 0.13 mm

### Data collection

**Table:**

Bruker SMART CCD diffractometer	1468 independent reflections
Radiation source: sealed tube	1140 reflections with *I* > 2σ(*I*)
Graphite monochromator	*R*_int_ = 0.031
φ and ω scans	θ_max_ = 25.1°, θ_min_ = 2.5°
Absorption correction: multi-scan (*SADABS*; Bruker, 2010)	*h* = −18→17
*T*_min_ = 0.880, *T*_max_ = 0.958	*k* = 0→11
5726 measured reflections	*l* = 0→13

### Refinement

**Table:**

Refinement on *F*^2^	Primary atom site location: structure-invariant direct methods
Least-squares matrix: full	Secondary atom site location: difference Fourier map
*R*[*F*^2^ > 2σ(*F*^2^)] = 0.037	Hydrogen site location: inferred from neighbouring sites
*wR*(*F*^2^) = 0.110	H-atom parameters constrained
*S* = 1.05	*w* = 1/[σ^2^(*F*_o_^2^) + (0.0553*P*)^2^ + 1.0043*P*] where *P* = (*F*_o_^2^ + 2*F*_c_^2^)/3
1468 reflections	(Δ/σ)_max_ = 0.001
101 parameters	Δρ_max_ = 0.21 e Å^−^^3^
0 restraints	Δρ_min_ = −0.18 e Å^−^^3^

### Special details

**Table:**

Refinement. Refinement of *F*^2^ against all reflections. The weighted *R*-factor *wR* and goodness of fit *S* are based on *F*^2^, conventional *R*-factors *R* are based on *F*, with *F* set to zero for negative *F*^2^. The threshold expression of *F*^2^ > 2σ(*F*^2^) is used only for calculating *R*-factors(gt) *etc*. and is not relevant to the choice of reflections for refinement. *R*-factors based on *F*^2^ are statistically about twice as large as those based on *F*, and *R*- factors based on all data will be even larger.

### Fractional atomic coordinates and isotropic or equivalent isotropic displacement parameters (Å^2^)

**Table:**

	*x*	*y*	*z*	*U*_iso_*/*U*_eq_	
S1	0.56058 (4)	0.49722 (6)	0.73116 (5)	0.0567 (2)	
O1	0.47025 (9)	0.69997 (17)	0.58995 (15)	0.0626 (5)	
N1	0.61329 (11)	0.65722 (19)	0.57979 (17)	0.0514 (5)	
C1	0.54116 (13)	0.6361 (2)	0.62131 (19)	0.0481 (5)	
C2	0.60771 (17)	0.7649 (3)	0.4905 (2)	0.0697 (7)	
H2A	0.5446	0.7887	0.4530	0.105*	
H2B	0.6341	0.7328	0.4301	0.105*	
H2C	0.6407	0.8438	0.5298	0.105*	
C3	0.69962 (13)	0.5872 (2)	0.62742 (19)	0.0466 (5)	
C4	0.76230 (16)	0.6332 (3)	0.7319 (2)	0.0681 (7)	
H4A	0.7486	0.7071	0.7739	0.082*	
C5	0.84668 (17)	0.5672 (4)	0.7739 (3)	0.0852 (9)	
H5A	0.8893	0.5962	0.8451	0.102*	
C6	0.86714 (18)	0.4597 (3)	0.7107 (3)	0.0806 (9)	
H6A	0.9235	0.4159	0.7393	0.097*	
C7	0.80540 (18)	0.4168 (3)	0.6063 (3)	0.0716 (7)	
H7A	0.8202	0.3449	0.5631	0.086*	
C8	0.72070 (16)	0.4796 (2)	0.5640 (2)	0.0562 (6)	
H8A	0.6782	0.4493	0.4932	0.067*	

### Atomic displacement parameters (Å^2^)

**Table:**

	*U*^11^	*U*^22^	*U*^33^	*U*^12^	*U*^13^	*U*^23^
S1	0.0465 (3)	0.0663 (4)	0.0681 (4)	0.0096 (3)	0.0337 (3)	0.0104 (3)
O1	0.0407 (8)	0.0691 (10)	0.0836 (12)	0.0153 (7)	0.0269 (8)	0.0072 (8)
N1	0.0395 (9)	0.0581 (11)	0.0632 (11)	0.0094 (8)	0.0255 (8)	0.0135 (9)
C1	0.0387 (11)	0.0530 (12)	0.0566 (13)	0.0036 (9)	0.0205 (9)	−0.0051 (10)
C2	0.0644 (15)	0.0712 (16)	0.0834 (18)	0.0110 (13)	0.0370 (14)	0.0239 (14)
C3	0.0349 (10)	0.0546 (12)	0.0573 (13)	0.0039 (9)	0.0244 (9)	0.0078 (10)
C4	0.0496 (13)	0.0865 (18)	0.0723 (16)	0.0010 (13)	0.0244 (12)	−0.0099 (14)
C5	0.0468 (14)	0.120 (3)	0.0796 (19)	−0.0082 (16)	0.0043 (13)	0.0141 (19)
C6	0.0438 (14)	0.092 (2)	0.114 (2)	0.0204 (14)	0.0361 (16)	0.0376 (19)
C7	0.0624 (15)	0.0610 (15)	0.105 (2)	0.0186 (13)	0.0467 (16)	0.0171 (15)
C8	0.0508 (13)	0.0567 (14)	0.0675 (14)	0.0032 (10)	0.0274 (11)	0.0033 (11)

### Geometric parameters (Å, º)

**Table:**

S1—O1^i^	3.0078 (18)	C3—C8	1.377 (3)
S1—C1	1.825 (2)	C4—C5	1.393 (4)
S1—S1^i^	2.0262 (11)	C4—H4A	0.9300
O1—C1	1.209 (2)	C5—C6	1.371 (4)
N1—C1	1.345 (3)	C5—H5A	0.9300
N1—C3	1.442 (2)	C6—C7	1.359 (4)
N1—C2	1.461 (3)	C6—H6A	0.9300
C2—H2A	0.9600	C7—C8	1.384 (3)
C2—H2B	0.9600	C7—H7A	0.9300
C2—H2C	0.9600	C8—H8A	0.9300
C3—C4	1.376 (3)		
			
C1—S1—S1^i^	100.51 (7)	C3—C4—C5	118.9 (3)
C1—N1—C3	122.98 (17)	C3—C4—H4A	120.5
C1—N1—C2	118.97 (17)	C5—C4—H4A	120.5
C3—N1—C2	117.79 (17)	C6—C5—C4	120.3 (3)
O1—C1—N1	124.8 (2)	C6—C5—H5A	119.8
O1—C1—S1	122.58 (16)	C4—C5—H5A	119.8
N1—C1—S1	112.64 (14)	C7—C6—C5	120.3 (2)
N1—C2—H2A	109.5	C7—C6—H6A	119.8
N1—C2—H2B	109.5	C5—C6—H6A	119.8
H2A—C2—H2B	109.5	C6—C7—C8	120.3 (3)
N1—C2—H2C	109.5	C6—C7—H7A	119.8
H2A—C2—H2C	109.5	C8—C7—H7A	119.8
H2B—C2—H2C	109.5	C3—C8—C7	119.6 (2)
C4—C3—C8	120.5 (2)	C3—C8—H8A	120.2
C4—C3—N1	119.9 (2)	C7—C8—H8A	120.2
C8—C3—N1	119.5 (2)		
			
C3—N1—C1—O1	174.2 (2)	C8—C3—C4—C5	−1.3 (4)
C2—N1—C1—O1	0.2 (3)	N1—C3—C4—C5	−177.6 (2)
C3—N1—C1—S1	−6.4 (3)	C3—C4—C5—C6	1.0 (4)
C2—N1—C1—S1	179.66 (17)	C4—C5—C6—C7	0.2 (4)
S1^i^—S1—C1—O1	−0.1 (2)	C5—C6—C7—C8	−1.2 (4)
S1^i^—S1—C1—N1	−179.48 (15)	C4—C3—C8—C7	0.4 (3)
C1—N1—C3—C4	−81.5 (3)	N1—C3—C8—C7	176.7 (2)
C2—N1—C3—C4	92.5 (3)	C6—C7—C8—C3	0.9 (4)
C1—N1—C3—C8	102.2 (3)	C1—S1—S1^i^—C1^i^	−81.55 (14)
C2—N1—C3—C8	−83.8 (3)		

Symmetry code: (i) −*x*+1, *y*, −*z*+3/2.
